# Parents’ knowledge, attitude and practice regarding childhood circumcision: a cross-sectional study in the central region of Sichuan, China

**DOI:** 10.3389/fped.2025.1465998

**Published:** 2025-04-24

**Authors:** Bing Zou, Chunlin Tan, Rui Deng, Qiang Chen, Qiu Peng, Dong Xu, Yunpeng Gou, Yong Du, Kangming Li, Zhili Chen, Ping Yang

**Affiliations:** ^1^ Department of Pediatric Surgery, Suining Central Hospital, Suining, China; ^2^ Department of Urology, Affiliated Hospital of North Sichuan Medical College, Nanchong, China

**Keywords:** male circumcision, knowledge, attitude, practice, redundant prepuce, cross-sectional study

## Abstract

**Introduction:**

This cross-sectional study aimed to explore parents’ knowledge, attitudes, and practices (KAP) regarding male circumcision (MC) for their children.

**Methods:**

A cross-sectional study was conducted among parents of male children under 18 years old in the central region of Sichuan, China, between September 2023 and January 2024 Questionnaires were distributed to collect demographic information and KAP scores.

**Results:**

A total of 497 valid questionnaires were collected. The average knowledge score was 6.66 ± 2.34 (range: 0–10), attitude score was 38.04 ± 4.52 (range: 12–60), and practice score was 24.81 ± 3.49 (range: 6–30). Parents demonstrated highest awareness regarding the positive effects of MC on penis health, but showed lowest understanding of MC benefits for children with redundant prepuce or phimosis. Notably, 75% of parents indicated a willingness to have the procedure performed in the future. Multivariate analysis identified knowledge, attitude, and residence as independent factors associated with consent for childhood circumcision. Path analysis revealed positive influences from knowledge to attitude, knowledge to practice, and attitude to practice.

**Conclusions:**

The study highlights the importance of targeted educational interventions to enhance parental understanding and decision-making regarding childhood MC. Educational programs can empower parents to make well-informed decisions considering both cultural and medical contexts.

## Introduction

Male circumcision (MC) is a surgical procedure, which involves the removal of the foreskin covering the head of the penis (1). MC has been associated with potential health benefits, such as a lower risk of suffering urinary tract infections, sexually transmitted infections, and penile cancer (2). Additionally, MC promotes better genital hygiene, eliminates phimosis, and prevents balanitis (3). On the other hand, potential drawbacks, including pain, discomfort, and possible complications such as bleeding, infection, and penile damage also related to this procedure (4). Furthermore, ethical considerations arise when MC is performed on children, raising questions about the appropriateness of performing a non-therapeutic surgical procedure in children without their consent (5, 6). Some argue that performing MC on minors without medical necessity may infringe upon a child's bodily autonomy and right to make informed choices about their own health. However, an alternative ethical perspective asserts that parents have a duty to act in the best interest of their children, particularly in cases where medical procedures can offer long-term health benefits. In this view, parents are entrusted with making medical decisions that maximize the probability of good health for their children, especially when the child is not yet mature enough to make an informed decision independently (7, 8).

MC is a prevalent procedure globally, with approximately one-third of males undergoing circumcision due to religious, cultural, and medical reasons (9). However, only 5% of Chinese males are circumcised (10), and when performed, it is typically for therapeutic reasons, such as treating phimosis or recurrent balanitis, rather than for prophylactic purposes (11). Typically, MC is conducted during infancy or childhood, particularly in regions such as the Middle East, Africa, and North America (12). In the United States, circumcision rates have declined in recent years but are still relatively high, with around 60%–70% of newborn boys being circumcised (13, 14). This decline is largely attributed to the growing Hispanic population, which traditionally does not practice circumcision. When accounting for demographic shifts, circumcision rates have remained relatively stable among non-Hispanic populations, including African Americans, indicating that general attitudes and practices toward MC in the US have not significantly changed despite opposition from anti-circumcision groups (15). Conversely, in China, where circumcision rates are notably lower, a 2012 study revealed a prevalent reluctance among parents to opt for early infant male circumcision due to concerns regarding pain and procedural risks (16). However, there has been a gradual increase in the number of Chinese parents choosing circumcision for their sons, particularly in larger cities (16), indicating a shifting trend in cultural acceptance or medical considerations.

In the United States, most newborn circumcisions are performed by OB-GYNs before hospital discharge, while some are conducted by pediatricians shortly after discharge. Additionally, Jewish newborn males are traditionally circumcised on the eighth day after birth by a mohel, a ritual circumcisor. The American Academy of Pediatrics (AAP) states that the health benefits of newborn male circumcision outweigh the risks, justifying access to this procedure for families who choose it, rather than issuing a universal recommendation for all male infants. The American College of Obstetricians and Gynecologists has endorsed this stance. Given the emphasis on informed decision-making in these guidelines, parents or guardians should be educated about the potential risks and benefits of MC and make a decision that is in the child's best interest (17). Additionally, the child's values and preferences should be considered as they mature enough to participate in decision-making. Thus, it would be valuable to conduct a Knowledge, Attitudes, and Practices (KAP) study (18) among parents of children under 18 years old in China to understand their perceptions and behaviors regarding MC, given the lack of such studies within the Chinese population.

This study aimed to explore parents’ KAP regarding MC for children in the central region of Sichuan, China. We hypothesize that the findings will identify knowledge gaps and reveal parental attitudes and beliefs about MC, facilitating the development of targeted educational programs and effective public health policies and interventions for children in the central region of Sichuan, China.

## Materials and methods

### Study design and participants

A cross-sectional anonymous survey was conducted in the central region of Sichuan, China, between September 2023 and January 2024, including parents of male children aged 0–18 years old. The Ethics Review Committee for Medical Research of Suining Central Hospital approved this study (approval number: KYLLK20230024), and all participants provided informed consent.

Inclusion criteria were the following: being a parent (18–55 years old) of a male child less than 18 years old. Exclusion criteria included: individuals with a response time of <2 min and individuals who did not complete the questionnaire.

### Questionnaire

The questionnaire was designed by the authors based on previous studies (7, 19). A pilot study was conducted among 54 participants, with a Cronbach's *α* = 0.878, which exhibited good internal consistency.

The final questionnaire comprised four dimensions: demographic information (age, gender, residence, ethnicity, education, occupation, monthly income, age, and health insurance type of the child), knowledge, attitude, and practice. The knowledge dimension had 11 questions, each scored as 1 for a correct response and 0 for an incorrect or uncertain response, with a total score range of 0–10. Question 2 was descriptive and not scored, and in question 4, “wrong” was considered correct based on medical classification. The attitude dimension consisted of 12 statements assessed on a 5-point Likert scale, ranging from 5 (“strongly agree”) to 1 (“strongly disagree”), with a total score range of 12–60. Questions 4, 6, 7, 9, and 11 were reverse-scored to ensure consistency in interpretation. The practice dimension included 6 questions evaluating parents’ willingness to have their child circumcised, also scored on a 5-point Likert scale from 5 (“strongly agree”) to 1 (“strongly disagree”), with a total possible score ranging from 6 to 30. KAP was considered moderate when participants scored between 60%–80% of the total score and favorable when they scored above 80%. The questionnaire was provided in the Supplementary File.

This study utilized an online questionnaire created through Sojump (Changsha Ranxing Information Technology) made accessible via a link or QR code. The authors and their colleagues distributed the QR code to eligible parents visiting any outpatient department at Suining Central Hospital between September 2023 to January 2024. Participants could complete the questionnaire by scanning the QR code or clicking the link created online.

### Statistical analysis

Statistical analysis was performed using Stata 17.0 (Stata Corporation, College Station, TX, USA). All continuous variables were conformed to a normal distribution and were compared using independent-samples *t*-test or one-way ANOVA. The Pearson correlation analysis was used to analyze the relationship between knowledge, attitude, and practice scores. Multivariate logistic regression analysis was conducted to identify independent variables associated with good practice. The multivariate logistic regression analysis included univariate variables with *p* < 0.05. Path analysis was performed to elucidate the relationships among knowledge, attitude, and practice. A two-sided *p* < 0.05 was considered statistically significant.

## Results

### Demographic characteristics

The present study involved 521 parent participants. Ten participants were excluded due to response times <120 s; nine were under 18 years old, three were over 55, and two had children older than 18. Consequently, 497 (95.39%) valid questionnaires were collected. The participants’ average age was 34, with males comprising 44.87% of the sample. The children's average age was 6 years old. Over half of the participants (51.11%) received education beyond junior college. The majority identified as Han Chinese (99.20%) and resided in urban areas (73.04%). Additionally, most participants did not have supplemental commercial medical insurance (76.66%) and did not have children who underwent male circumcision (69.82%) (Table 1).

**Table 1:** Demographic information of the participants.

Characteristics	*N* (%)	Knowledge		Attitude		Practice	
		Score	*P*	Score	*P*	Score	*P*
Total	497	6.66 ± 2.34		38.04 ± 4.52		24.81 ± 3.49	
Parent's age (years)	34 (23, 51)		0.552		0.662		0.057
≤34	282 (56.74)	6.60 ± 2.38		38.09 ± 4.13		25.02 ± 3.61	
>34	215 (43.26)	6.73 ± 2.29		37.98 ± 5.00		24.54 ± 3.32	
Parent's gender			0.172		0.059		0.892
Male	223 (44.87)	6.76 ± 2.37		38.37 ± 4.12		24.74 ± 3.68	
Female	274 (55.13)	6.57 ± 2.33		37.77 ± 4.81		24.87 ± 3.33	
Child's age (years)	6 (0, 18)		0.513		0.462		0.246
≤6	259 (52.11)	6.75 ± 2.24		37.76 ± 4.05		24.97 ± 3.31	
>6	238 (47.89)	6.55 ± 2.46		38.35 ± 4.98		24.65 ± 3.68	
Residence			<0.001		0.512		<0.001
Urban	363 (73.04)	6.94 ± 2.13		38.06 ± 4.62		25.17 ± 3.38	
Nonurban	134 (26.96)	5.88 ± 2.69		37.99 ± 4.27		23.85 ± 3.61	
Ethnicity			0.019		0.094		0.410
Han	493 (99.20)	6.68 ± 2.34		38.06 ± 4.53		24.80 ± 3.50	
Other	4 (0.80)	4.25 ± 1.26		35.25 ± 1.26		26.00 ± 2.16	
Education			<0.001		0.008		0.044
Secondary school and below	243 (48.89)	6.21 ± 2.49		38.51 ± 3.97		24.47 ± 3.70	
Junior college and above	254 (51.11)	7.08 ± 2.12		37.59 ± 4.96		25.14 ± 3.25	
Occupation			<0.001		0.072		0.129
Regular employee	213 (42.86)	7.03 ± 2.17		37.61 ± 4.97		25.13 ± 3.31	
Non-regular employee^a^	145 (29.18)	6.78 ± 2.24		38.35 ± 3.75		24.54 ± 3.51	
Other	139 (27.97)	5.96 ± 2.56		38.37 ± 4.51		24.60 ± 3.72	
Monthly income (yuan)			<0.001		0.084		<0.001
<5,000	209 (42.05)	6.18 ± 2.51		37.50 ± 4.15		24.14 ± 3.25	
5,000–10,000	185 (37.22)	7.09 ± 2.11		38.66 ± 4.90		25.19 ± 3.65	
>10,000	103 (20.72)	6.83 ± 2.25		38.03 ± 4.45		25.50 ± 3.25	
With or without additional commercial medical insurance			0.007		0.097		0.003
Yes	116 (23.34)	7.19 ± 1.99		38.74 ± 4.97		25.63 ± 3.36	
No	381 (76.66)	6.49 ± 2.42		37.83 ± 4.36		24.56 ± 3.49	
Having children undergoing male circumcision			0.096		<0.001		0.591
Yes	150 (30.18)	6.96 ± 2.10		39.57 ± 4.98		24.82 ± 3.42	
No	347 (69.82)	6.52 ± 2.43		37.38 ± 4.15		24.81 ± 3.53	

^a^ Part-time employee or freelance.

### Parental KAP on MC

The average scores were 6.66 ± 2.34 (66.6% of the total, moderate), 38.04 ± 4.52 (63.4% of the total, moderate), and 24.81 ± 3.49 (82.7% of the total, good) for knowledge, attitude, and practice dimensions, respectively (Table 1). Parents demonstrated the highest awareness (90.34%) regarding the positive effects of MC on penis health. However, they showed the lowest understanding (8.25%) of the potential benefits of MC for children with redundant prepuce or phimosis. The question concerning whether phimosis is considered a disease was not scored, as the answer remains inconclusive. Based on the responses, 55.53% of parents considered it a disease, 13.68% did not, and 30.78% were uncertain (Supplementary Table S1). Parents living in urban areas (*p* < 0.001), of Han ethnicity (*p* = 0.019), with higher education (*p* < 0.001), regular employment (*p* < 0.001), higher income (*p* < 0.001), and additional commercial health insurance (*p* = 0.007) were found to have a better knowledge (Table 1).

**Figure 1:**
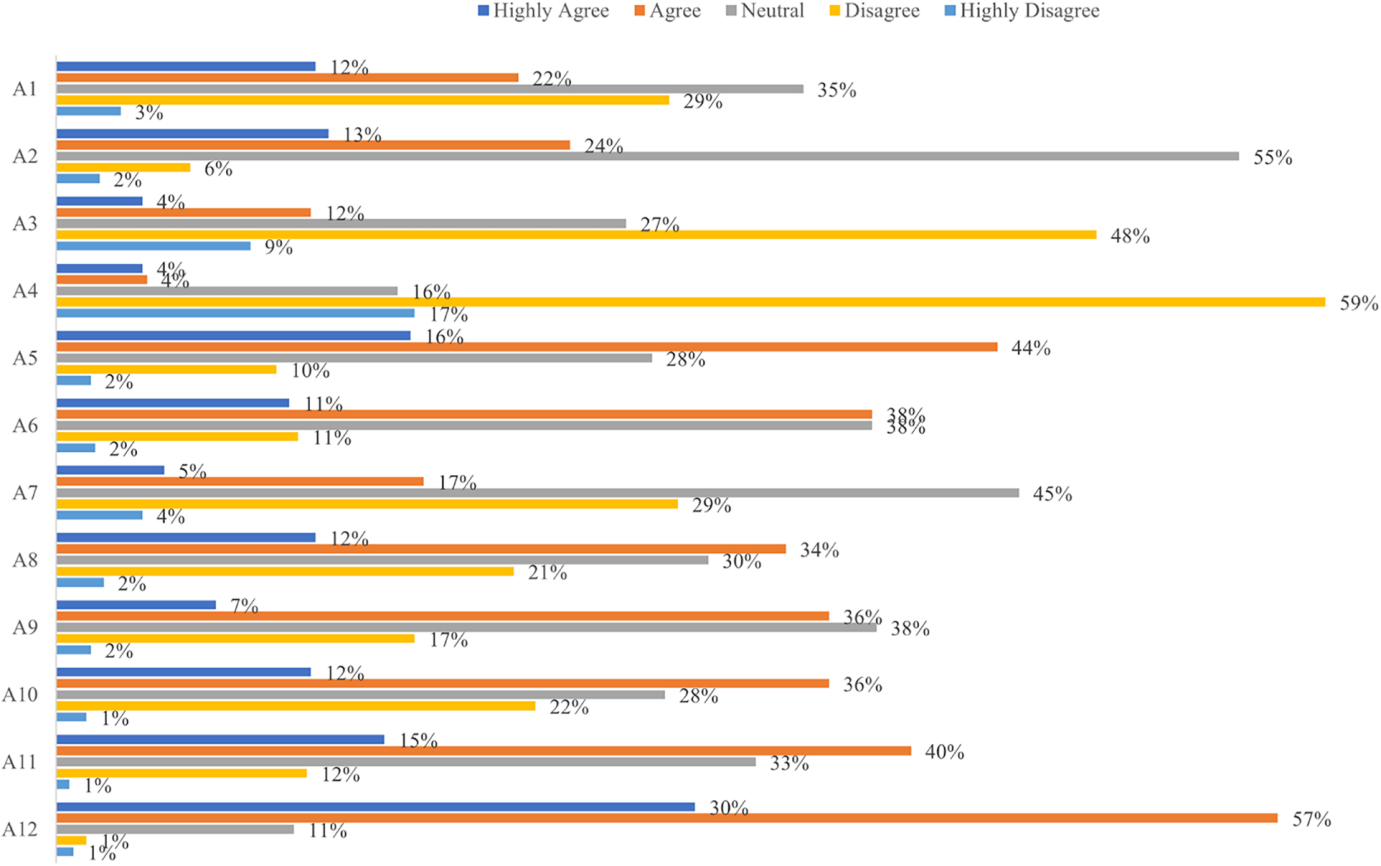
Distribution of responses in the attitude assessment. A1: Circumcision is necessary for all boys; A2: I have a full understanding of male circumcision; A3: My child may face peer pressure or teasing if he is uncircumcised; A4: My child may face peer pressure or teasing if he is circumcised; A5: Male circumcision can improve the aesthetic appearance of the penis; A6: My child is fearful of undergoing male circumcision; A7: Male circumcision can be a painful procedure; A8: Male circumcision is required for proper childhood growth and development; A9: I am concerned about potential complications associated with male circumcision; A10: Male circumcision may have better outcomes when performed at a younger age; A11: Male circumcision is a private medical procedure; A12: It is important to monitor for potential complications following a child's male circumcision procedure.

In the attitude assessment, parents expressed the most supportive attitude towards monitoring potential complications after a circumcision procedure and the issue of peer pressure or teasing related to circumcision (Figure 1). Parents with lower education (*p* = 0.008) and those having children who underwent MC (*p* < 0.001) had more supportive attitudes towards MC (Table 1). While overall attitude scores between male and female participants did not show a statistically significant difference (*p* = 0.059), further analysis revealed gender-based differences in certain specific attitude items. Notably, males showed significantly greater agreement with statements regarding circumcision necessity (A1, *p* = 0.034), understanding of MC (A2, *p* < 0.001), and belief that circumcision should be performed at a younger age (A10, *p* = 0.012). Regarding the reverse-scored question A4, females showed significantly higher scores (*p* < 0.001), indicating they were less concerned about potential teasing if a child is circumcised compared to male participants (Supplementary Table S2).

**Figure 2:**
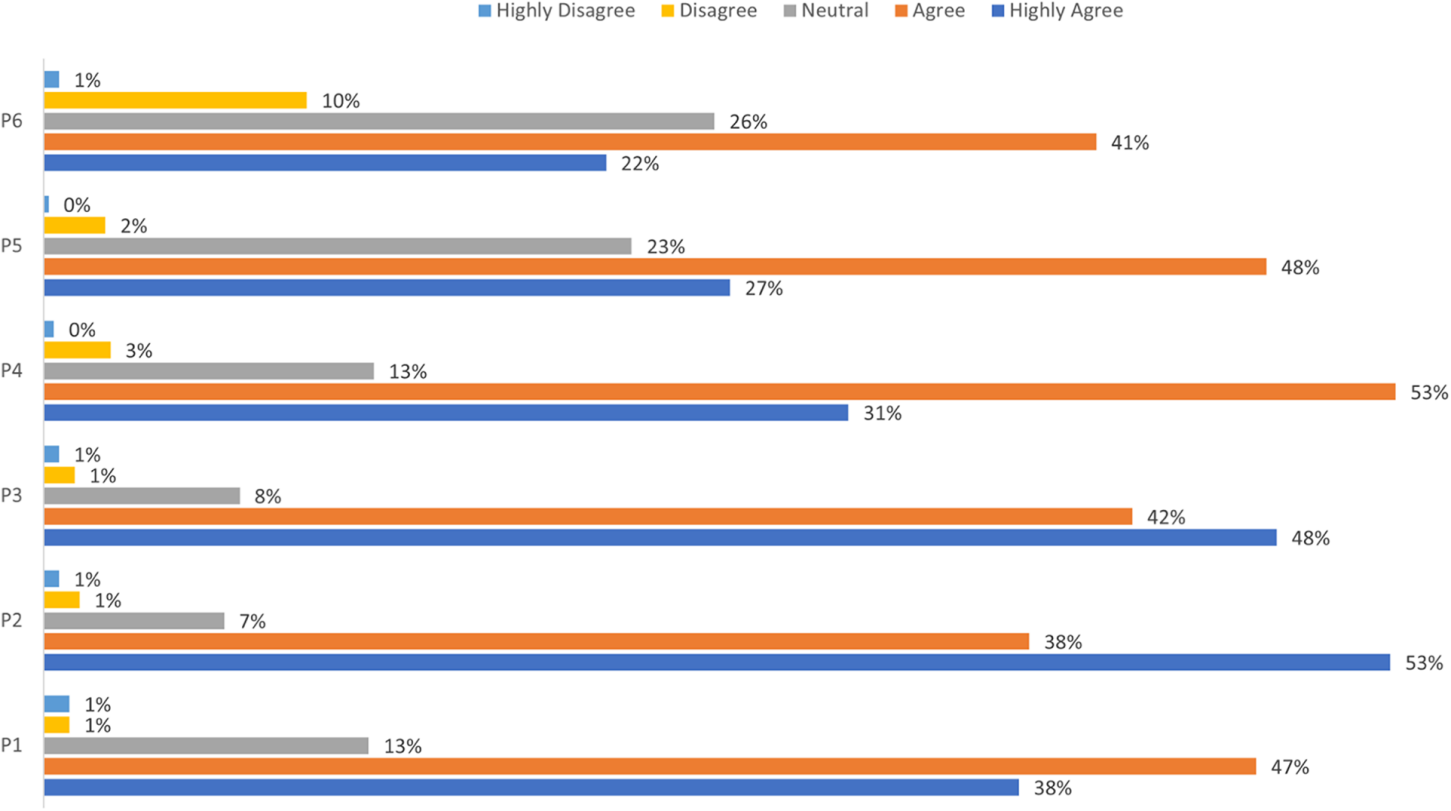
Distribution of responses in the practice assessment. P1: I will ensure that I have a complete understanding of all relevant information before my child undergoes circumcision; P2: I prefer that my child undergoes male circumcision in a large public hospital; P3: I will have open communication with my child regarding the procedure if I decide to have him circumcised; P4: If my child is hesitant about undergoing male circumcision, I will persuade him to do so; P5: I am considering having my child undergo circumcision; P6: If my child wishes to undergo male circumcision, I believe it is better for him to do so at a young age.

The practice assessment results indicated that parents supported MC, as evidenced by a high percentage of parents (75%) planning to have the procedure done in the future (Figure 2). In addition, correlation analysis revealed that knowledge scores were positively correlated with attitude (*r* = 0.345, *p* < 0.001) and practice scores (*r* = 0.434, *p* < 0.001), and attitude scores were positively correlated with practice scores (*r* = 0.440, *p* < 0.001) (Table 2).

**Table 2:** Correlation analysis for the knowledge, attitude, and practice dimension.

Variable	Knowledge	Attitude	Practice
Knowledge	1		
Attitude	0.345*	1	
Practice	0.414*	0.440*	1

* *P* < 0.001.

### Factors that independently associated with parental agreement for childhood MC

The results of the multivariate logistic analysis indicated that knowledge [OR = 1.21, 95% CI: (1.09, 1.34), *p* < 0.001], attitude [OR = 1.16, 95% CI: (1.10, 1.22), *p* < 0.001], and place of residence [OR = 0.54, 95% CI: (0.33, 0.90), *p* = 0.0171] were independent variables associated with parental agreement for childhood MC (Table 3).

**Table 3:** Multivariate logistic regression analysis of practice.

Characteristics	Univariate analysis		Multivariate analysis	
	OR (95%CI)	*P*	OR (95%CI)	*P*
Knowledge	1.40 (1.25, 1.52)	<0.001	1.21 (1.09, 1.34)	<0.001
Attitude	1.18 (1.12, 1.24)	<0.001	1.16 (1.10, 1.22)	<0.001
Parent's age (years)				
≤34	Ref.			
>35	0.72 (0.51, 1.03)	0.075		
Parent's gender				
Male	Ref.			
Female	0.96 (0.67, 1.36)	0.813		
Child's age (years)				
≤6	Ref.		Ref.	
>7	0.70 (0.49, 1.00)	0.050	0.73 (0.49, 1.10)	0.135
Residence				
Urban	Ref.		Ref.	
Non-urban	0.38 (0.25, 0.58)	<0.001	0.54 (0.33, 0.90)	0.017
Education				
Secondary school and below	Ref.		Ref.	
Junior college and above	1.70 (1.19, 2.42)	0.003	1.10 (0.66, 1.83)	0.710
Occupation				
Regular employee	Ref.		Ref.	
Non-regular employee^a^ =	0.61 (0.40, 0.94)	0.024	0.70 (0.39, 1.23)	0.215
Other	0.52 (0.34, 0.81)	0.003	0.77 (0.44, 1.36)	0.372
Monthly income (Yuan)				
<5,000	Ref.		Ref.	
5,000–10,000	2.30 (1.53, 3.44)	<0.001	1.57 (1.00, 2.47)	0.050
>10,000	2.35 (1.45, 3.80)	0.001	1.67 (0.97, 2.90)	0.065
With or without additional commercial medical insurance				
No	Ref.		Ref.	
Yes	1.98 (1.29, 3.03)	0.002	1.27 (0.78, 2.06)	0.336
Having children receiving male circumcision				
No	Ref.			
Yes	0.75 (0.51, 1.11)	0.147		

^a^ Part-time employee or freelance.

### Pathway analysis of parental KAP in childhood MC

**Figure 3:**
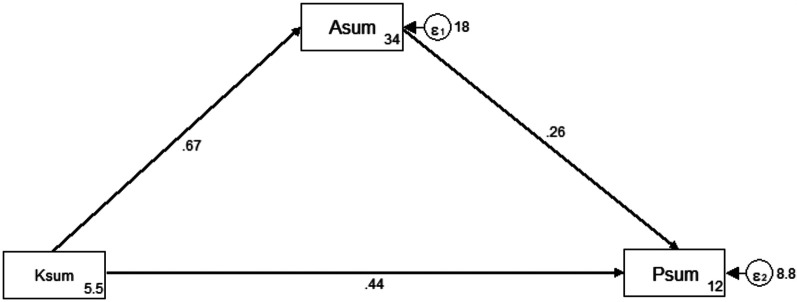
Path analysis.

To elucidate the relationship between parents’ knowledge, attitude, and practice regarding their children's circumcision, we performed a path analysis. As shown in Figure 3; Table 4, we observed direct positive influences from knowledge to attitude (path coefficient = 0.67, *p* < 0.001), knowledge to practice (path coefficient = 0.44, *p* = 0.002), and attitude to practice (path coefficient = 0.26, *p* < 0.001), along with an indirect positive influence from knowledge to practice (path coefficient = 0.17, *p* < 0.001). These results suggest a sequential relationship wherein parents’ knowledge positively influences their attitude, which in turn, positively influences their practice regarding children's circumcision.

**Table 4:** Path analysis.

Model paths	Total effects		Direct Effect		Indirect effect	
	β (95% CI)	*P*	β (95% CI)	*P*	β (95% CI)	*P*
Asum <-						
Ksum	0.66 (0.50,0.82)	<0.001	0.66 (0.50,0.82)	<0.001		
Psum <-						
Asum	0.26 (0.19,0.32)	<0.001	0.26 (0.19,0.32)	<0.001		
Ksum	0.61 (0.49,0.73)	0.002	0.44 (0.32,0.56)	<0.001	0.17 (0.11,0.23)	<0.001

## Discussion

The present study demonstrated that parents had moderate knowledge and attitudes, and exhibited an inclination towards MC for their children. Knowledge, attitudes, and place of residence were independent factors that affected practice scores. The study emphasized the need for improved education on the benefits and drawbacks of circumcision. Given that parental reluctance toward early infant circumcision is often driven by concerns about pain and procedural risks, targeted educational efforts should address the advantages of early infancy as the optimal timing for circumcision, including lower procedural risks, no need for stitches, and faster healing.

Phimosis refers to the condition in which retracting the penis foreskin becomes difficult. Two types of phimosis (physiologic and pathologic) can complicate the diagnosis (20). Physiologic phimosis is temporary, often seen in 2–4-year-olds, and typically resolves on its own as the foreskin becomes more elastic. Pathologic phimosis, on the other hand, is caused by balanitis xerotica obliterans, a skin condition characterized by scarring and tissue damage, similar to lichen sclerosis histologically (21). As a result, the second question in the knowledge domain was not included in the computation and was treated as a descriptive outcome. The fact that 30.78% of participants were uncertain about whether phimosis is considered a disease suggests that this may be a complex or ambiguous issue for some people. The uncertainty may stem from a lack of understanding of the condition or a lack of consensus on its classification as a disease.

This study found that parents living in urban areas with higher education levels, stable employment, higher incomes, and additional commercial health insurance exhibited a better comprehension of MC. Similarly, a questionnaire survey in Hong Kong found that misconceptions about phimosis and circumcision were more prevalent among parents from lower socio-economic classes (22). Moreover, our study revealed that parents with lower education levels and those whose children underwent MC displayed more supportive attitudes towards MC. This finding suggests that prior experience with MC may contribute to a more supportive attitude toward the procedure. Additionally, although overall attitude scores between male and female participants were not significantly different, we found gender-based differences in specific attitude items. Male participants exhibited stronger agreement with statements regarding circumcision necessity and benefits, while also more concerned about social implications such as potential teasing. These findings highlight potential gender-based variations in parental perceptions of circumcision and underscore the importance of addressing these differences in educational interventions. Interestingly, our study also found that parents living in rural areas were less likely to opt for circumcision than those in urban areas. This result implies that cultural and regional differences may influence a person's decision to practice MC. In contrast to our study, a nationwide questionnaire study conducted in Korea revealed that parents with higher education and socioeconomic status tended to have more favorable attitudes towards circumcision. Additionally, the present study found no significant difference in responses between parents living in urban and rural areas (23). These findings suggest that cultural and societal factors may vary across countries, and further research is needed to understand the factors shaping attitudes toward MC.

The study also identified a positive correlation between knowledge, attitude, and practice. Parents who better understood male circumcision and had more supportive attitudes toward it were more likely to have their children circumcised. Our path analysis further revealed a sequential relationship where parents’ knowledge positively influenced their attitude, which in turn, positively influenced their practice concerning children's circumcision, with additional positive influences observed from knowledge to practice. Our results align with prior research emphasizing the pivotal role of parental understanding and beliefs in shaping healthcare-related decisions for their children (24). Furthermore, the pathway from knowledge to practice highlights the potential impact of parental education programs in guiding healthcare behaviors (25). Notably, the correlation between attitudes and practices underscores the importance of addressing parental perceptions and beliefs to facilitate the translation of supportive attitudes into diverse healthcare actions (26). Improving parental knowledge and attitudes through education could potentially influence the practice of MC (27, 28). However, a survey of 190 women who had given birth to healthy male infants in Texas, US, found that parental education about the medical indications and possible risks of circumcision did not influence the decision-making process regarding neonatal circumcision (29). As a result, the impact of parental education on the practice of MC may be influenced by various contextual and cultural factors.

In addition to knowledge and attitudes, the place of residence has been identified as a significant factor influencing parents’ practices related to MC. Comparative studies reveal variations in MC practices across different regions and cultures. For example, in Ethiopia, traditional MC practices vary notably with the place of residence (30). Urban residents in Turkana County, Kenya, tend to be more aware of the protective effects of circumcision, and social stigma from being uncircumcised plays a significant role in influencing the practice (31). Conversely, male rural-to-urban migrants in Western China exhibit lower acceptance rates of circumcision compared to the general population in China or worldwide. These observations highlight the need for culturally and regionally tailored approaches in any educational interventions related to circumcision.

The present study has some limitations. Firstly, the results are based on self-reported data and may be subject to recall bias or social desirability bias. Secondly, most participants were Han Chinese living in urban areas, which limits the generalizability of the findings to other populations with different sociodemographic backgrounds, such as rural or ethnic minority communities. Thirdly, this study was conducted among parents in the central region of Sichuan, China, and the findings may not be generalizable to other populations or contexts. Additionally, while we analyzed differences in attitudes between male and female participants, we did not collect data on attitude differences between fathers and mothers within the same couple, which remains a limitation. Finally, it may be beneficial to consider enhancing survey neutrality, particularly in the knowledge section, which could lead to more robust data collection and facilitate the derivation of meaningful conclusions.

In conclusion, this study identified a significant association between parental knowledge, attitudes, and practices concerning childhood MC. The findings indicate a sequential relationship wherein enhanced knowledge about MC tends to improve parental attitudes, which in turn may influence their practices. These results underscore the importance of comprehensive educational programs that inform parents about MC, enabling them to make informed decisions based on a clear understanding of potential benefits and risks.

## Data Availability

The original contributions presented in the study are included in the article/Supplementary Material, further inquiries can be directed to the corresponding author.
