# Supplementary material for: Parents’ knowledge, attitude and practice regarding childhood circumcision: a cross-sectional study in the central region of Sichuan, China
Source: Front Pediatr. 2025 Apr 24;13:1465998. doi: 10.3389/fped.2025.1465998 (PMC12058898; doi:10.3389/fped.2025.1465998)
Supplement: Supplementary file 1 [file Supplementaryfile1.docx]

| Dear participants,  We are researchers at XXX Hospital and would like to invite you to participate in our study. The purpose of this study is to better understand parents' knowledge, attitudes, and practices regarding male circumcision in their children. Your participation in this study is entirely voluntary, and we hope that more people may benefit from its results in the future.  If you agree to participate, please follow the instructions below:  Please answer the questionnaire as honestly as possible, based on your unique circumstances. There are no absolute right or wrong answers to any of the questions. Please submit your responses by the given deadline.  Your personal information, such as gender and age, will be collected as part of the study. However, we will keep this information strictly confidential and will not disclose it to any third party. Participation in this study will not have any adverse effects on you.  As a participant, you will have access to information and research progress related to this study at any time. If you decide to withdraw from the study, please inform us so that we can exclude your data from the analysis.  Thank you for considering our invitation to participate in this study. Your contribution is greatly appreciated and will help support our scientific research efforts.  □ After being informed, I provided consent for the collected data to be used for scientific research purposes.  Informed Consent Signature：  Date of participation： year month day | | | | | | | | | |  |
| --- | --- | --- | --- | --- | --- | --- | --- | --- | --- | --- |
| **Part I Basic Information** | | | | | | | | | |  |
| **1. Gender：** | a. Male | | | | b. Female | | | | |  |
| **2. Gge：** | years old | | | | | | | | |  |
| **3. Your child's age：** | years old | | | | | | | | |  |
| **4. Residence：** | a. Rural  b. Urban  c. Suburb | | | | | | | | |  |
| **5.**  **Ethnicity：** | a. Han  b. Minorities  If you belong to a minority group, your ethnicity is： | | | | | | | | |  |
| **6****. Education：** | a. Primary School and below  b. Middle School/High School/Technical secondary school  c. Junior College/Bachelor’s degree  d. Master’s degree and above | | | | | | | | |  |
| **7.**  **Occupation:** | a. Regular employee  b. Part-time  c. Freelance  d. Unemployed  e. Full-time housewife/husband  f. Other | | | | | | | | |  |
| **8. Household income per capita:______ Yuan** | a. < 2000  b. 2000–5000  c. 5000–10000  d. 10000–20000  e. > 20000 | | | | | | | | |  |
| **9. Your child's medical insurance type: (Multiple Choice)** | a. Basic medical insurance for urban & rural residents  b. Commercial insurance  c. No insurance | | | | | | | | |  |
| **10. Have you had your child circumcised?** | a. Yes  b. No | | | | | | | | |  |
| **Part II Knowledge** | | | | | | | | |  |  |
| **1. All men have a prepuce.** | a. Correct | | b. Wrong | | | | c. Uncertain | | |  |
| **2. Do you consider redundant prepuce or phimosis a medical condition that requires treatment?** | a. Correct | | b. Wrong | | | | c. Uncertain | | |  |
| **3. Redundant prepuce or phimosis can lead to inflammation or cancer of the penile head foreskin.** | a. Correct | | b. Wrong | | | | c. Uncertain | | |  |
| **4. For children with redundant prepuce or phimosis, male circumcision is one potential treatment option.** | a. Correct | | b. Wrong | | | | c. Uncertain | | |  |
| **5. Male circumcision promotes penis development.** | a. Correct | | b. Wrong | | | | c. Uncertain | | |  |
| **6. Male circumcision improves penis health.** | a. Correct | | b. Wrong | | | | c. Uncertain | | |  |
| **7. Male circumcision prevents HIV infection.** | a. Correct | | b. Wrong | | | | c. Uncertain | | |  |
| **8. Male circumcision prevents the infection of the penis or urinary tract.** | a. Correct | | b. Wrong | | | | c. Uncertain | | |  |
| **9. Male circumcision prevents the spouse from developing gynecological diseases.** | a. Correct | | b. Wrong | | | | c. Uncertain | | |  |
| **10. Anesthesia is required during male circumcision procedures.** | a. Correct | | b. Wrong | | | | c. Uncertain | | |  |
| **11. Male circumcision may be associated with potential complications, such as bleeding, infection, and pain.** | a. Correct | | b. Wrong | | | | c. Uncertain | | |  |
| **Part III Attitudes** | | | | | | | | | | |
| **1. I believe circumcision is beneficial for all boys regardless of their medical condition.** | a. Strongly agree | b. Agree | | c. Neutral | | d. Disagree | | e. Strongly disagree | | |
| **2. I have a full understanding of male circumcision.** | a. Strongly agree | b. Agree | | c. Neutral | | d. Disagree | | e. Strongly disagree | | |
| **3. My child may face peer pressure or teasing if he is uncircumcised.** | a. Strongly agree | b. Agree | | c. Neutral | | d. Disagree | | e. Strongly disagree | | |
| **4. My child may face peer pressure or teasing if he is circumcised.** | a. Strongly agree | b. Agree | | c. Neutral | | d. Disagree | | e. Strongly disagree | | |
| **5. Male circumcision can improve the aesthetic appearance of the penis.** | a. Strongly agree | b. Agree | | c. Neutral | | d. Disagree | | e. Strongly disagree | | |
| **6. My child is fearful of undergoing male circumcision.** | a. Strongly agree | b. Agree | | c. Neutral | | d. Disagree | | e. Strongly disagree | | |
| **7. Male circumcision can be a painful procedure.** | a. Strongly agree | b. Agree | | c. Neutral | | d. Disagree | | e. Strongly disagree | | |
| **8. Male circumcision is required for proper childhood growth and development.** | a. Strongly agree | b. Agree | | c. Neutral | | d. Disagree | | e. Strongly disagree | | |
| **9. I am concerned about potential complications associated with male circumcision.** | a. Strongly agree | b. Agree | | c. Neutral | | d. Disagree | | e. Strongly disagree | | |
| **10. Male circumcision may have better outcomes when performed at a younger age.** | a. Strongly agree | b. Agree | | c. Neutral | | d. Disagree | | e. Strongly disagree | | |
| **11. Male circumcision is a private medical procedure.** | a. Strongly agree | b. Agree | | c. Neutral | | d. Disagree | | e. Strongly disagree | | |
| **12. It is important to monitor for potential complications following a child's male circumcision procedure.** | a. Strongly agree | b. Agree | | c. Neutral | | d. Disagree | | e. Strongly disagree | | |
| **Part IV Practices** | | | | | | | | | | |
| **1. I will ensure that I have a complete understanding of all relevant information before my child undergoes circumcision.** | a. Strongly agree | b. Agree | | c. Neutral | | d. Disagree | | e. Strongly disagree | | |
| **2. I prefer that my child undergoes male circumcision in a large public hospital.** | a. Strongly agree | b. Agree | | c. Neutral | | d. Disagree | | e. Strongly disagree | | |
| **3. I will have open communication with my child regarding the procedure if I decide to have him circumcised.** | a. Strongly agree | b. Agree | | c. Neutral | | d. Disagree | | e. Strongly disagree | | |
| **4. If my child is hesitant about undergoing male circumcision, I will persuade him to do so.** | a. Strongly agree | b. Agree | | c. Neutral | | d. Disagree | | e. Strongly disagree | | |
| **5. I plan to have my child undergo circumcision in the future.** | a. Strongly agree | b. Agree | | c. Neutral | | d. Disagree | | e. Strongly disagree | | |
| **6. If my child wishes to undergo male circumcision, I believe it is better for him to do so at a young age.** | a. Strongly agree | b. Agree | | c. Neutral | | d. Disagree | | e. Strongly disagree | | |
