# Supplementary figures and images for: Parents’ knowledge, attitude and practice regarding childhood circumcision: a cross-sectional study in the central region of Sichuan, China
Source: Front Pediatr. 2025 Apr 24;13:1465998. doi: 10.3389/fped.2025.1465998 (PMC12058898; doi:10.3389/fped.2025.1465998)

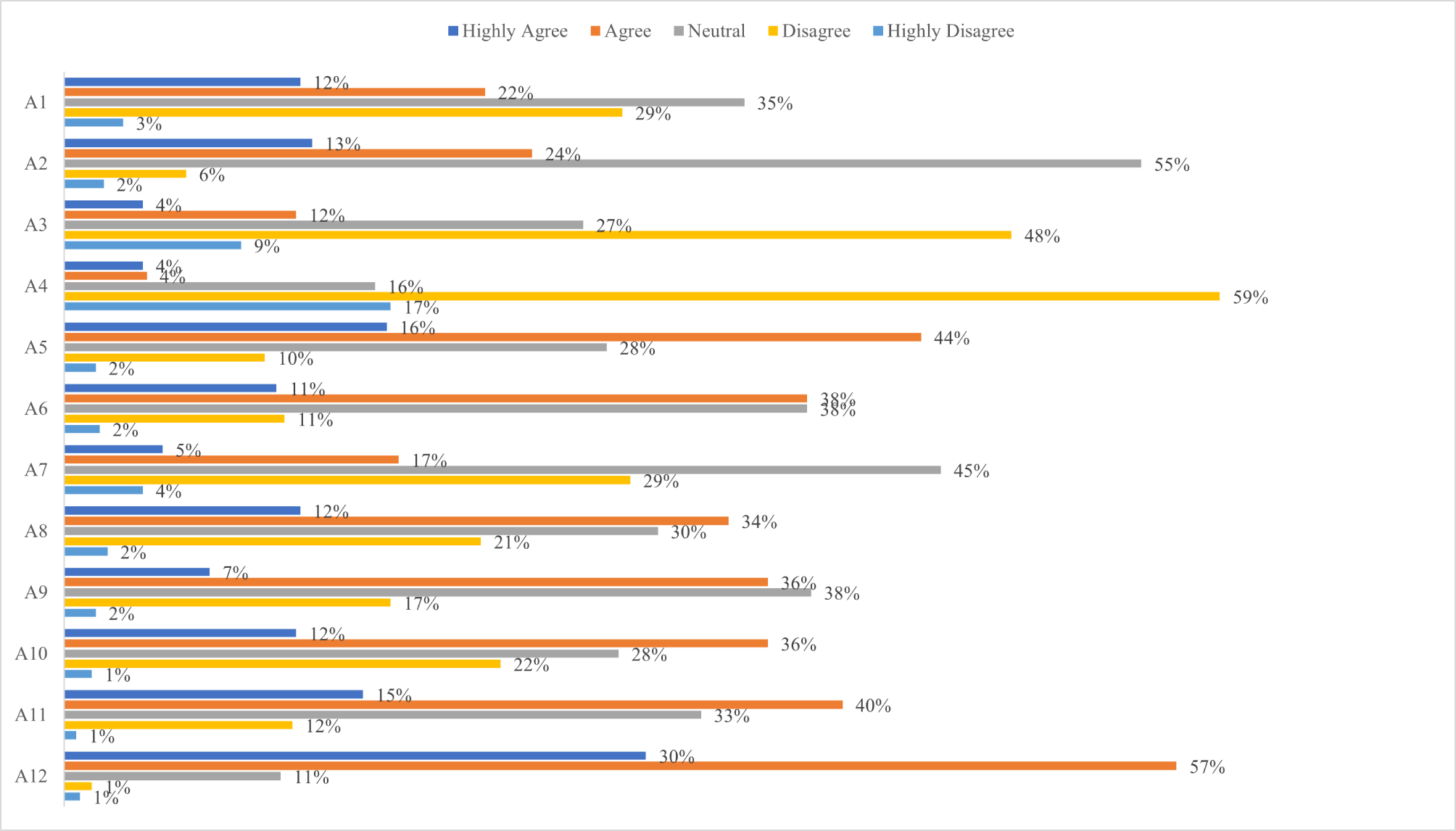

Supplement: Supplementary file 2 [file Image1.tiff]

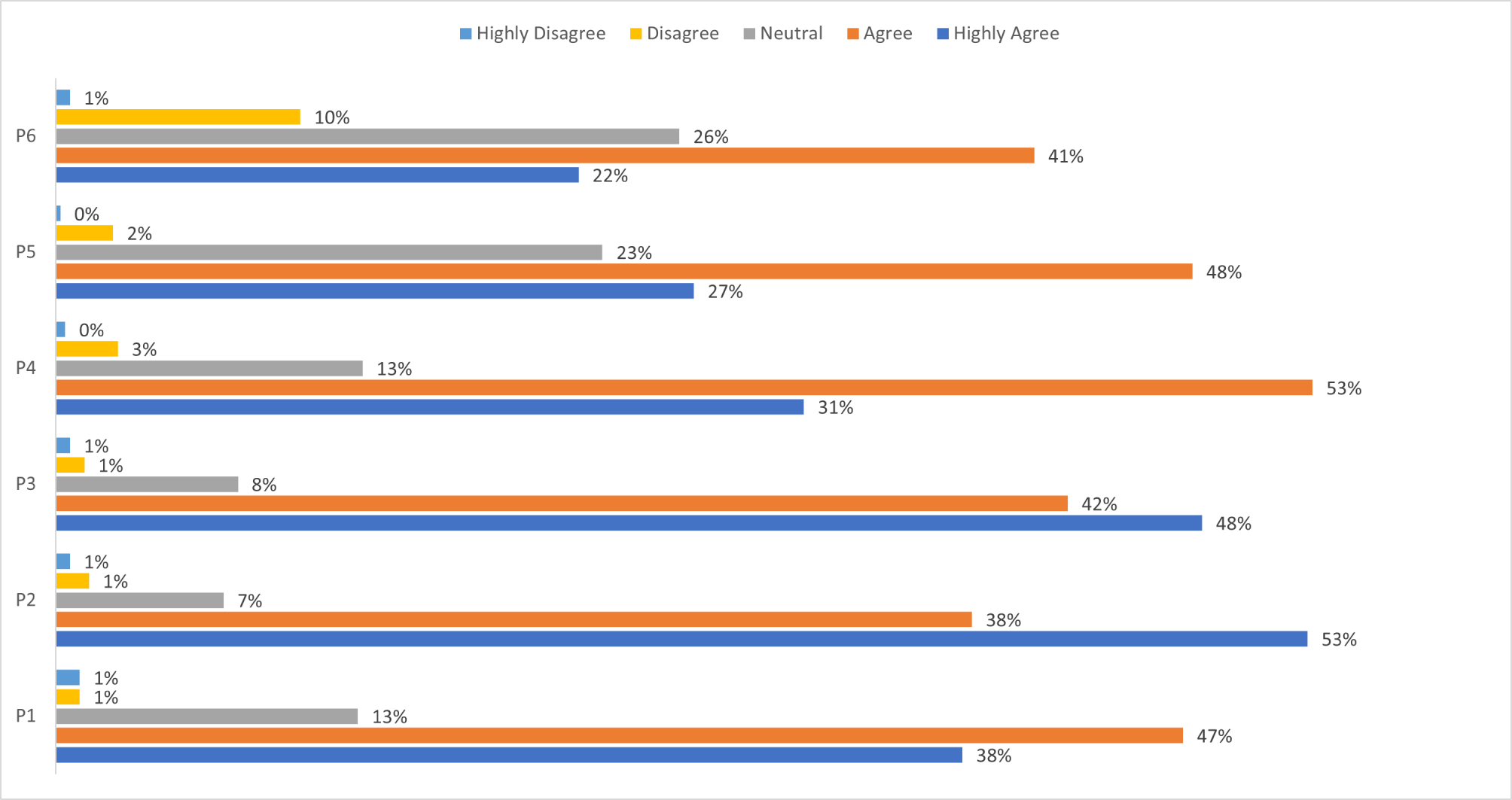

Supplement: Supplementary file 3 [file Image2.tiff]
