# Supplementary material for: Parents’ knowledge, attitude and practice regarding childhood circumcision: a cross-sectional study in the central region of Sichuan, China
Source: Front Pediatr. 2025 Apr 24;13:1465998. doi: 10.3389/fped.2025.1465998 (PMC12058898; doi:10.3389/fped.2025.1465998)
Supplement: Supplementary file 4 [file Table1.docx]

Table S1. Response of the knowledge dimension

| Knowledge | Correctness rate, n (%) |
| --- | --- |
| All men have a prepuce. | 193 (38.83) |
| Redundant prepuce or phimosis can lead to inflammation or cancer of the penile head foreskin. | 341 (68.61) |
| Male circumcision may be beneficial for children with redundant prepuce or phimosis. | 41 (8.25) |
| Male circumcision promotes penis development. | 364 (73.24) |
| Male circumcision improves penis health. | 449 (90.34) |
| Male circumcision prevents HIV infection. | 306 (61.57) |
| Male circumcision prevents the infection of the penis or urinary tract. | 417 (83.90) |
| Male circumcision prevents the spouse from developing gynecological diseases. | 411 (82.70) |
| Anesthesia is required during male circumcision procedures. | 401 (80.68) |
| Male circumcision may be associated with potential complications, such as bleeding, infection, and pain. | 385 (77.46) |

Table S2. Comparison of individual attitude items between male and female participants.

| Attitude | **Male** | **Female** | **P** |
| --- | --- | --- | --- |
|  | **Mean±SD** | **Mean±SD** |  |
| Circumcision is necessary for all boys | 3.22±1.05 | 3.01±1.03 | 0.034 |
| I have a full understanding of male circumcision | 3.56±0.81 | 3.24±0.87 | <0.001 |
| My child may face peer pressure or teasing if he is uncircumcised | 2.61±0.97 | 2.46±0.93 | 0.074 |
| My child may face peer pressure or teasing if he is circumcised | 3.57±0.98 | 3.98±0.78 | <0.001 |
| Male circumcision can improve the aesthetic appearance of the penis | 3.69±0.89 | 3.58±0.95 | 0.295 |
| My child is fearful of undergoing male circumcision | 2.54±0.89 | 2.55±0.89 | 0.955 |
| Male circumcision can be a painful procedure | 3.03±0.93 | 3.14±0.87 | 0.376 |
| Male circumcision is required for proper childhood growth and development | 3.42±0.97 | 3.24±1.03 | 0.052 |
| I am concerned about potential complications associated with male circumcision | 2.63±0.90 | 2.73±0.88 | 0.341 |
| Male circumcision may have better outcomes when performed at a younger age | 3.47±0.98 | 3.24±0.99 | 0.012 |
| Male circumcision is a private medical procedure | 2.44±0.92 | 2.40±0.89 | 0.761 |
| It is important to monitor for potential complications following a child's male circumcision procedure | 4.12±0.74 | 4.14±0.69 | 0.959 |
